# Nanocarrier-Mediated Topical Insulin Delivery for Wound Healing

**DOI:** 10.3390/ma14154257

**Published:** 2021-07-30

**Authors:** Ana S. Macedo, Francisca Mendes, Patrícia Filipe, Salette Reis, Pedro Fonte

**Affiliations:** 1 Associated Lab for Green Chemistry (LAQV), REQUIMTE, Department of Chemical Sciences—Applied Chemistry Lab, Faculty of Pharmacy, University of Porto, Rua de Jorge Viterbo Ferreira 228, 4050-313 Porto, Portugal; anatmacedo@hotmail.com (A.S.M.); shreis@ff.up.pt (S.R.); 2 Faculdade de Ciências, Universidade de Lisboa, 1749-016 Lisboa, Portugal; franciscarmendes@gmail.com; 3 Center of Marine Sciences (CCMAR), University of Algarve, Gambelas Campus, 8005-139 Faro, Portugal; patricia.cfilipe@gmail.com; 4 Department of Chemistry and Pharmacy, Faculty of Sciences and Technology, University of Algarve, Gambelas Campus, 8005-139 Faro, Portugal; 5 Department of Bioengineering, iBB—Institute for Bioengineering and Biosciences, Instituto Superior Técnico, Universidade de Lisboa, Av. Rovisco Pais, 1049-001 Lisboa, Portugal; 6 Associate Laboratory i4HB—Institute for Health and Bioeconomy at Instituto Superior Técnico, Universidade de Lisboa, Av. Rovisco Pais, 1049-001 Lisboa, Portugal

**Keywords:** insulin, wound healing, nanomedicine, topical delivery, wounds, burns, polymer nanoparticle, lipid nanoparticle, inorganic nanoparticle

## Abstract

Wound care has been clinically demanding due to inefficacious treatment that represents an economic burden for healthcare systems. In Europe, approximately 7 million people are diagnosed with untreated wounds, leading to a cost between 6.000€ and 10.000€ per patient/year. In the United States of America, 1.5 million people over 65 years old suffer from chronic wounds. A promising therapeutic strategy is the use of exogenous growth factors because they are decreased at the wound site, limiting the recovery of the skin. Insulin is one of the cheapest growth factors in the market able to accelerate the re-epithelialization and stimulate angiogenesis and cell migration. However, the effectiveness of topical insulin in wound healing is hampered by the proteases in the wound bed. The encapsulation into nanoparticles improves its stability in the wound, providing adhesion to the mucosal surface and allowing its sustained release. The aim of this review is to perform a standing point about a promising strategy to treat different types of wounds by the topical delivery of insulin-loaded nanocarriers.

## 1. Introduction

Wound healing is a biological and dynamic process, in which the skin begins to self-repair after an injury. The prevalence of wounds has been increasing, becoming a significant public health issue with high morbidity and mortality rates among the population. About 100 million people across the globe suffer from acute wounds and 300 million from chronic wounds, representing a major cost for health-care systems. Populations with high-risk of incidence of chronic wounds, such as diabetics, obese, and elderly people, have a high probability of experiencing it during their lifetime [1]. While the treatment of acute and superficial wounds is highly efficacious, treating chronic wounds is challenging [2]. The approach for chronic wound healing has been in reducing the inflammation and topical application of exogenous growth factors. However, ongoing therapies lack efficacy, leading to long-term recovery due to excessive proteases at the wound site [3]. Insulin, a peptide hormone used in diabetic patients to manage blood glucose levels is also used as a growth factor, being able to mitigate the compromised skin by triggering cell migration and proliferation in order to coalesce and heal the wound. Insulin stimulates the migration of keratinocytes, influences the proliferation of fibroblasts and the production of extracellular matrix proteins, and modulates the release of inflammatory cytokines [4]. Moreover, insulin is also a low-cost growth factor and a compatible wound dressing, turning the inclusion of this peptide hormone into dressing matrices beneficial for wound treatment efficacy [5].

Despite the benefits of insulin in wound healing, its lack of stability in the wound bed due to the proteases action hampers its therapeutic benefits. In past years, research in the field of nanomedicine has progressed toward improving nanomaterials features, such as large surface area to volume ratio, antimicrobial activity, or electric conductivity of small-scale particles. Many biological processes occur in the nanometer range, causing the nanoparticles (NPs) to be singular instruments for drug delivery [6]. Thus, the encapsulation of insulin into nanoparticles is an excellent strategy to improve the stability of the protein in the wound bed and deliver it in a controlled manner, improving the therapeutic outcome. Different materials have been explored to produce insulin-loaded nanocarriers, mainly from lipid, polymer, or inorganic sources.

The main aim of this review is to address the benefits of nanocarrier-mediated topical insulin delivery as a promising strategy for wound healing applications. Herein, the effect of insulin in wound healing and the advantages of its encapsulation into nanocarriers are addressed. More importantly, the different types of nanocarriers used to deliver insulin topically as well as their in vitro and in vivo performance will be discussed. Finally, pipeline products for growth factors delivery and toxicity concerns are regarded. It is expected that this contribution may be a good starting point to future research works in the field.

## 2. Anatomophysiology of Skin

### 2.1. Healthy Skin

**Figure 1:**
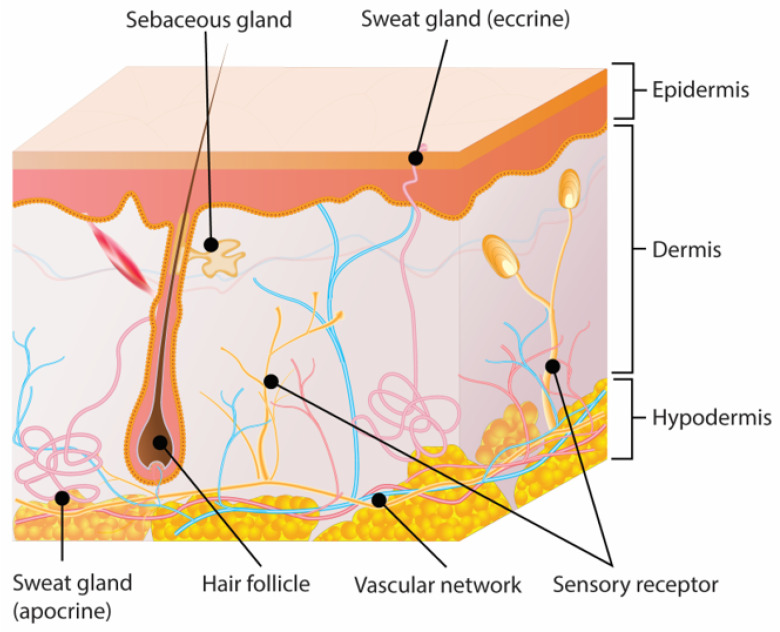
Histological structure of skin.

The skin is the largest organ of the body, covering its whole surface with a surface area between 1.5–2.0 m² of an average adult [4]. The skin, composed of epidermis, dermis, and hypodermis (Figure 1), is a vital barrier between the environment and the body against pathogens, lesions, loss of water and heat, capable of offering sensory perception and immune protection [5]. The primary barrier of the skin is in the upper layer, namely the stratum corneum. The stratum corneum is formed by corneocytes or terminally differentiated keratinocytes inserted in the extracellular lipid matrix formed by ceramides, cholesterol, and fatty acids. Underlying the stratum corneum are the stratum granulosum, stratum spinosum, and stratum basale from the epidermis [7]. The stratum corneum building blocks, keratinocytes, are influenced by cytokines, which play key roles for inflammatory processes, including cell signaling, consenting cell secretion, division, migration, growth and repair. Also present in the epidermis are the Langerhans cells, which are formed in the bone marrow [8].

Underlying the epidermis is the dermis consisting of two sublayers: the reticular dermis and the papillary dermis. The reticular dermis is composed of connective tissue proteins and a vascular network. The papillary dermis is composed of capillary loops essential for oxygenation and nutrient supply, sebaceous glands, sweat glands, nerves, and hair follicles [9]. The cells in the dermis are fibroblasts, mast cells, histiocytes, adipocytes, and macrophages [10]. Gay et al. showed that fibroblasts stimulate type I collagen synthesis, offering support to the skin [11]. In 2005, Lee & Cho demonstrated interactions between skin fibroblasts and human epidermal keratinocytes by increasing the expression of type IV collagen, type VII collagen, and laminin [12]. Finally, the hypodermis is located at the bottom layer of the skin, between the dermis and the muscle. The hypodermis is composed of connective tissue, blood vessels, nerves and adipocytes, serving as the body energy reserve and protection against mechanical injury of the body from mechanical injuries [13].

### 2.2. Skin Wounds

**Figure 2:**
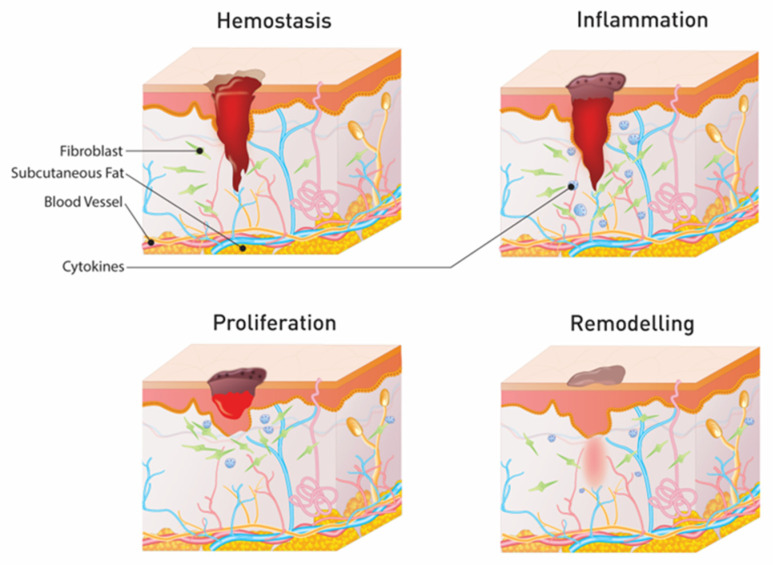
Stages of wound healing of skin.

The wound is a consequence of an intentional, accidental, or disease process causing damage to the skin barrier [14]. Wound healing is a well-ordered and multi-stage process in which different cells interact with the extracellular matrix (ECM) to promote skin regeneration. Upon injury, cytokines are released, and hemostasis begins, progressing to the phases of inflammation, proliferation, and remodeling (Figure 2) [15]. After completing hemostasis and preventing the blood hemorrhage, the inflammation process occurs. Arteriolar constriction causes a decrease in blood flow, leading to a reflex vasodilatation and relaxation of the arterial vessels. The inflammatory response contributes to the onset of prostaglandin synthesis, which results in local vasodilation. Mast cells release histamine to increase vasodilatation and vascular permeability, facilitating the movement of inflammatory cells and proteins into the extracellular space around the wound [16]. Other inflammatory mediators such as kinin and serotonin are released, boosting microvascular permeability. The proliferative phase is characterized by the proliferation of fibroblasts and collagen deposition. A new matrix will be composed of fibronectins, proteoglycans, and collagen. The angiogenesis process begins, followed by the proliferation of epithelial cells and the formation of new connective tissue and blood vessels in the wound bed. Macrophages and platelets secrete platelet-derived growth factor (PDGF), which induces fibroblast proliferation and chemotaxis. Fibroblasts migrate to the wound and move through the extracellular matrix, linking fibrin, vitronectin, and fibronectin, via an arginine-glycine-aspartic acid amino acid sequence. Furthermore, fibroblasts produce matrix metalloproteinases (MMPs), which remove impaired matrix components and enable fibroblast movement. Tumor necrosis factor-alpha (TNF-α) and basic fibroblast growth factor (bFGF) activate endothelial cells to initiate angiogenesis. Angiogenesis is the physiological process that allows the formation of new blood vessels to replace the damaged capillaries, providing adequate oxygen and nutrients to the wound area. Low oxygen levels, low pH, and high lactate levels also stimulate the vascularization process [17]. The final phase of wound healing is the remodeling phase, in which novel epithelium is developed together with scar formation. In acute wounds, the levels of collagen increases, promoting wound tensile strength [18]. Regular collagen formation and disintegration, as well as remodeling of the extracellular matrix, are completed within 3 weeks of trauma until a steady shape is reached. The approximation of the wound edges and the connective tissue size reduction is a consequence of the interactions between the extracellular matrix and the fibroblasts.

Overall, the process of wound healing consists of three key mechanisms: set of granulation tissue, contraction, and epithelialization. Depending on the type of wound, these three mechanisms act in different ways. Chronic wounds, namely pressure ulcers, diabetic ulcers, and venous ulcers, are wounds that have a prolonged inflammation and do not heal within 12 weeks (Table 1) [19]. These wounds have high levels of MMPs, an increase of inflammatory cytokines release, debris, and infection [20]. As the microbial load on the wound expands, healing is considerably interrupted. The inflammatory response releases an excess amount of reactive oxygen species and inflammatory cytokines, leading to a continuous state of inflammation and proteolytic enzymes. Increased levels of protease in the wound result in the deterioration of local proteins such as extracellular matrix proteins and growth factors, matrix component reduction, epithelialization process disturbance, and wound contraction failure [21,22]. Thus, chronic wounds are characterized by excessive inflammation, hypoxia, high biological bioburden, prolonged protease activity, neuropathy and, in cases of irreparable tissue damage, fibrosis. Essentially, once chronic inflammation is stagnant or devitalized tissue is surgically removed, the therapy for chronic wounds prevents infection and promotes healing in the same way as the acute wounds [23].

### 2.3. Burns

**Figure 3:**
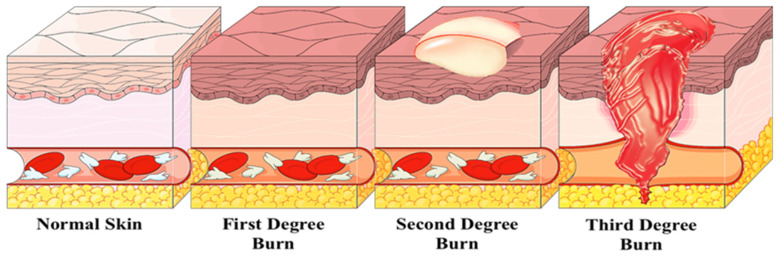
First to third degrees of burn wounds in human skin.

A burn is defined as a coagulative destruction of the skin and can be superficial or thick with moderate to heavy extension. Partially thick burn wounds typically form epithelial cell coverage over a period of no more than 20 days, whereas full-thickness burn wounds may require the removal of damaged and dead tissue to improve healing [32]. Thermal damage, radiation, electricity, and chemicals can cause burns. Skin recovery and various manifestations such as respiratory failure, infection, shock, or electrolyte variation are based on body area and burn depth. The burn extent is expressed as a percentage of the total body surface area (% TBSA) [33]. Burns present high mortality and morbidity levels. In the United States, burns are the seventh leading cause of death from all unintentional injuries [34]. Burn treatment costs are around USD 7.5 billion/year, adding treatment for infection, inflammation, and ischemia [35]. Each year in the United Kingdom, approximately 250.000 people attend hospitals due to burns [36]. Burns are categorized into four types, depending on the depth of the dermal lesion (Figure 3). First-degree burns involve damage to the superficial epidermis and manifest redness, dryness, and hypersensitivity. In second-degree burns, there is complete elimination of the epidermis and partial dermal loss. The skin turns red and edematous with blisters. Third-degree burns are full-thickness burns that reach the hypodermis. Full-thickness burns are, in most cases, painless, as a result of nerve damage [33]. Finally, fourth-degree burns are burns extended up to the bone or muscle. Overall, the efficacy of treatment relies on its extent [37].

Increased capillary permeability induced by burns leads to leakage of small protein molecules and edema [38]. The inflammatory response is triggered, activating the synthesis of inflammatory mediators, such as prostaglandins, leukotrienes, tumor necrosis factor (TNF), nitric oxide, or histamine. In an early stage, histamine induces the venules to contract and microvascular permeability. Burned patients have shown inhibition of insulin release, stimulation of cortisol release, proteolysis, and gluconeogenesis [39].

## 3. Effect of Insulin in Wound Healing

### 3.1. Diabetic Wounds

Patients suffering from diabetes mellitus manifest several complications such as nephropathy, neuropathy, or retinopathy. In wound healing, cell migration, growth and differentiation are also absent among diabetic patients. The low levels of growth factors, fibroblast dysfunction, increased proteolytic activity, and impairment in collagen assembly are manifested in diabetic wounds. Moreover, diabetic patients show increased levels of TNF-α and IL-6, leading to severe inflammation and insulin resistance [40,41].

Peripheral neuropathy causes chronic wounds in the limbs, commonly referred to as diabetic foot ulcer, lower extremity arterial disease, and foot deformity [42]. It is prevalent in about 7% of diabetic patients and is more recurrent among the elderly. Diabetic foot ulcer care involves a multifaceted procedure, consisting of surgical removal of the necrotic tissue, prevention or treatment of infections, limb elevation, and compression as well as revascularization [43].

In 1923, William Thalhimer was the first to describe the insulin activity in postoperative acidosis [44]. In parallel, Foster showed improvement in postoperative recovery among 20 diabetic patients receiving between 30 and 75 of insulin units intravenously. Wound healing among the diabetic patients was ameliorated and the number of deaths due to infection decreased approximately 30% in type I diabetic patients [45]. In a case study in 1965, a 56-year-old woman with diabetes was amputated below her knee due to gangrene on her foot and leg. The amputated stump became infected with *Streptococcus pyogenes*. She was administrated both cloxacillin and erythromycin, yet no improvement was observed. Thus, the researchers topically administered 20 IU of soluble insulin in gauze covered by a bandage, changing it twice a day. Four days later, the mitigation of the bacterial infection was observed [43]. Weringer tested the influence of insulin on wound recovery using diabetic and non-diabetic C57B1/6 mice treated and non-treated with insulin. The mice ears were perforated with a 0.1 cm dermal trephine and insulin was administered 40 h later. The ear wounds were evaluated by light and electron microscopy and fibroblasts, capillaries, polymorphonuclear leukocytes (PMN), collagen, and interstitial oedema were quantified by lineal point analysis. The untreated diabetic mice showed reduced capillaries, fibroblasts, collagen and PMN of total percent wound volume, in contrast to the similar response of both insulin-treated diabetic mice and control mice. The total percent wound volume for oedema was 44.0 ± 1.2% in the case of untreated diabetic mice, while insulin treated diabetic mice and control showed 13.5% ± 0.7 and 10.5 ± 1.3%, respectively. These results show that insulin treatment induced neovascularization, fibroblast proliferation, synthesis of collagen, and edema reduction in diabetic rats compared to placebo-treated diabetic and healthy rats [46].

Insulin pathways activation were analyzed in rats and diabetic patients with the administration of topical insulin cream [47]. The study compared male Wistar induced diabetic rats treated with and without topical insulin cream, along with non-diabetic rats. Wounds of diabetic rats were treated with 0.5 U/100 g of insulin cream or placebo, applied directly after skin excision and then every day until the end of the study. The authors observed an abrupt cellular response in diabetic animals and faster wound healing concomitant with an increase of protein levels, such as nitric oxide synthase (NOS) and vascular endothelial growth factor (VEGF), than the wounded tissue of normal rats. Conversely, the expression of insulin receptor substrate (IRS), protein kinase B (AKT), and extracellular signal–regulated kinases (ERK) was reduced in the wounded skin of diabetic rats, compared to the wounded skin of normal rats, indicating an increase of wound recovery period. Immunoblotting results showed approximately 25% of IRS-1 protein levels in the wounded skin of diabetic rats, in contrast to 270% of IRS-1 in the wounded skin of normal rats. AKT levels were approximately 50% less in the wounded skin of diabetic rats compared to the wounded skin of normal rats. The extracellular signal-regulated kinase (ERK) pathway was stimulated in the wound healing tissue on healthy rats, in contrast with undamaged skin, which highlights the major contribution that insulin signal transduction occupies in wound healing. In the clinical study, 22 diabetic patients with uncured lacerations for at least 3 months were randomized to assess the effect of topical insulin in a double-blind, placebo-controlled clinical trial for 8 weeks. Changes in the laceration length, width, and depth were observed. After 8 weeks, the patients that received the insulin cream showed a significant recovery in contrast to the patients that received the placebo cream. The treatment using insulin cream continued for both groups, and by the week 15, all patients showed total recovery.

Li and co-workers proposed an effective topical insulin delivery system to protect the insulin’s vulnerable structure by encapsulation into microparticles. Later, insulin microparticles were incorporated in a silk fibroin sponge. The authors were able to maintain the molecular structure of insulin and an extended release for 30 days. Moreover, the in vivo restorative effect of the matrix wound dressing was assessed in full-thickness diabetic wounds of Sprague-Dawley rats. After 3 weeks, histological analyses revealed strong cell relocation, collagen deposition, and epidermis, compared to control [48]. Ribeiro et al. prepared chitosan nanoparticles loaded with insulin embedded in a hydrogel and evaluated their therapeutic activity for wound healing in diabetic rats. The chitosan nanoparticles had a size below 200 nm, a positive potential zeta (33.7 ± 4.88 mV), and an AE of approximately 97%. In vivo studies showed the emergence of large blood vessels at day 7 for the animals treated with insulin-chitosan nanoparticles, although, empty and insulin-loaded chitosan nanoparticles were able to stimulate inflammatory cell proliferation and angiogenesis.

### 3.2. Non-Diabetic Wounds

Non-diabetic wounds are caused by trauma, pressure, inflammation, or cardiovascular disease without a diabetic origin. In comparison with diabetic wounds, non-diabetic wounds have shown less recurrence, lower incidence of infections, and less wound appearance in the lower limbs [49].

In 1930, the Barnet Joseph published a brief communication describing the effect of 10 IU of insulin administered once daily in five patients suffering from non-diabetic pressure ulcers. After 14 days, considerable improvement was observed in skin recovery [50]. Rosenthal, in 1968, used albino Wistar rats to evaluate the stimulation of wound healing caused by insulin. A laceration was performed on the abdomen of rats, and suspensions of protamine-zinc insulin were administered 3 days before the incision and every day thereafter. Insulin-treated animals expressed an increase in body weight and, in parallel, a 20% increase in skin toughness at the wound site [43].

Udupa and colleagues studied the histological aspects of insulin in skin wounds in rats. Albino rats, having an average weight of 110 g, were given 0.02 units of protamine zinc insulin per g bodyweight subcutaneously once per day through a period of 3 weeks. The trial was composed of 25 insulin-treated rats and 25 controls. Incision wounds in the abdominal of 50 mm in length were sutured with cotton stitches and the effects of wound reopening were observed. The insulin treated rats showed increased wound bursting strength in contrast to the control group, demonstrating a significant difference in the 7th and 12th days (*p* < 0.01). Histological evaluation showed accelerated collagen production and higher collagen structure organization, compared to the control group [51]. Additional studies observed the positive effect of insulin on wound recovery. Wilson and co-workers reported an 80-year-old patient suffering from a chronic postoperative abdominal wall wound who underwent negative-pressure wound therapy (NPWT) to improve wound closure, re-epithelialization, and humidity by promoting granulation tissue formation. However, after 21 days of NPWT, the chronic postoperative wound did not heal. The wound was washed with 2 IU/20 mL of human soluble insulin every day for 1 week. There was also no evidence of side effects regarding the use of insulin solution [52].

Zhang et al. (2007) questioned the efficacy of local insulin application in contrast with intravenous therapy concerning wound-healing rate, dropping of blood sugar, and potassium levels, as these signs appear in intravenous insulin administration. Insulin-zinc solution was injected locally around the wound and every other day into white male New Zealand rabbits. The authors noticed a higher healing rate among animals with localized insulin therapy compared to intravenous treatment cohorts [53]. Two years later, Liu et al. also studied the influence of insulin on keratinocyte migration and on PI3K-AKT signal transduction pathway activation in non-diabetic wounds. An in vitro study scratch assay was performed in cultured human keratinocytes, in which insulin treatment stimulated human keratinocyte migration and differentiation. The immunoblot test showed increased AKT activation levels after insulin application for at least 1 h. Furthermore, the in vivo study was composed of C57BL/6J mice with a 0.7 cm punch excision wound. Insulin was topically administered and animal lacerations exhibited a significant epithelium restoration compared to the control group [54].

### 3.3. Burns

The pathophysiology of burns differs from the biological mechanism for healing incisional and excisional wounds. The frequency of episodes of hyperglycemia and insulin resistance has been observed in patients with severe burns, decreasing the probability of wound healing. Studies have been conducted to obtain more information about the chemical processes involved during burning and on the effectiveness of its treatment with insulin [55]. In 1995, Sakurai et al. administered insulin intravenously to nine patients suffering from severe burns for 1 week to assess the variation in muscle protein levels. The novel amino acids production, their relocation from the blood, and a significant increase in muscle mass was observed due to the presence of insulin. Although the results were variable among patients, the authors observed that insulin treatment caused an increase of nearly 50% in protein synthesis at the wound site [56]. Later, in 1999, Zhang and collaborators considered the effect of insulin and growth hormone on protein synthesis in muscle and wounds. L-[ring-13C6]phenylalanine was used to determine protein anabolism. Rabbits which had undergone thermal ear burns were randomly divided into four groups: growth hormone, high-dose insulin, low-dose insulin, and blank control groups. Both high and low doses of insulin significantly decreased protein breakdown (*p* < 0.01), stimulating protein synthesis and inhibiting proteolysis, compared to the growth hormone group in which there was no change in protein balance [57]. Zhang and collaborators analyzed the protein levels in partial-thickness wounds through the introduction of insulin and several amino acids. Male New Zealand White rabbit ears were subjected to 72 °C water for 3 s. The scalded ears were used as an arteriovenous unit to measure the response of protein kinetics in the wound. The authors found increased wound protein production with both an exogenous amino acid mixture and high dose of insulin infusion administration, moving from 7 ± 4 μmol.100 g^−1^.h^−1^ to 1 ± 5 μmol.100 g^−1^.h^−1^ in the control group [58].

In 2001, Van den Berghe and co-workers evaluated the recovery rate of patients who received insulin at the surgical intensive care unit for one year. The authors conducted a prospective, randomized, controlled clinical trial where patients with assisted ventilation were divided into two groups. One group received a common insulin infusion therapy while the other experienced exhaustive insulin treatment. In the common insulin infusion group, glycemia was kept between 180 and 200 mg/dL, and insulin was only administered if the blood glucose level passed 215 mg/dL. In the exhaustive insulin therapy group, the glycemia was kept between 80 and 110 mg/dL. The authors verified that exhaustive insulin infusion reduced mortality by 8%, in contrast to 4.5% mortality reduction in the common insulin infusion group. Furthermore, patients who received exhaustive insulin treatment did not need extended assisted ventilation [59]. It has been suggested that severe burns cause insulin resistance along with impaired insulin signal. Following a severe burn, an acute phase reaction occurs in the liver, stimulating an inflammatory response and the immune system. In 2007, Jeschke et al. studied the impact of insulin therapy on severely burned hepatic-failure patients. The researchers observed improvement in liver function in burned patients treated with insulin by finding reduced levels of transcription factors and inflammatory cytokines. Insulin therapy increased recovery chances by minimizing sepsis in critically burned patients [60]. In 2010, Jeschke and collaborators explored the consequence of insulin administration on death rates in severely burned pediatric patients having burns above 30% of TBSA. The mortality rate for the control group was 11%, whereas the mortality rate for the pediatric insulin patients group was lower by 7% [61].

In 2012, Jeschke et al. developed a clinical study in severely burned children over 15 months to estimate the correlation between hyperglycemia and insulin resistance within unfolded protein response (UPR). UPR is a cellular stress response associated with endoplasmic reticulum (ER) stress. Researchers concluded that during patient rehabilitation, biochemical processes linked to the cell cycle, swelling, sarcoplasmic reticulum stress, and insulin resistance were altered [62]. Later, in 2013, Vural’s team examined the link between insulin and liver. The study was developed in severely burned patients who were receiving insulin. The authors found increased prealbumin and albumin levels, and decreased triglycerides and pro-inflammatory proteins levels with insulin therapy [63].

In 2010, Fram and co-workers found that insulin administration was safe and efficient in pediatric burn patients due to decrease of insulin resistance and improvement of metabolic hemostasis. A randomized clinical trial was conducted in burned pediatric patients whose burns exceeded 40% of TBSA. Two groups were created: conventional insulin treatment and intensive insulin treatment. The patients in the first group received an average insulin concentration of 55 µU/mL and had their blood glucose values below 215 mg/dL, while the second group received 105 µU/mL of insulin and had their blood glucose values between 80 and 110 mg/dL. Reduction of hepatic glucose secretion was lower in the conventional insulin treatment, in contrast to intensive insulin treatment, being 2.5 ± 0.6 vs. 5.0 ± 0.9 mg/kg·min, respectively. Intensive insulin treatment significantly improved mitochondrial oxidation of palmitate [64]. Tuvdendorj et al. performed skin transplants on burned children with more than 40% TBSA and examined the effect of insulin on the accelerating healing rate. In the first days after surgery, the authors observed an increase in the fractional synthetic rate (FSR) of the donor site wound protein, leading to increased collagen and laminin levels [65].

Azevedo et al. (2015) studied the effects of topical insulin application on wound healing in rats subjected to second-degree burns on the increase of collagen retention, stimulation of the microvascular network, and depression of the inflammatory phase. The animals were divided into four groups: diabetic rats receiving topical insulin cream, diabetic rats receiving placebo, healthy rats receiving topical insulin cream, and healthy rats receiving placebo. Histological examination showed increased Type III collagen levels between days 1 and 7 in healthy and diabetic wounds treated with insulin cream, compared to diabetic wounds treated with placebo. Angiogenesis and inflammatory responses were significantly higher at 2 weeks postburn in wounds treated with insulin cream to levels related to those of healthy rats receiving topical insulin cream and placebo [66]. Dhall et al. improved insulin stability required for wound healing by developing an alginate dressing containing insulin encapsulated in PLGA microparticles and showing sustained release for more than 3 weeks. The in vivo study was performed on the dorsum of Sprague–Dawley rats that had partial-thickness burn wounds made with a 15 mm diameter brass cylinder warmed up to 80 °C. Alginate dressings having 0.04 mg insulin/cm^2^ were then applied once every 3 days for 9 days, promoting higher levels of neutrophils and M1 macrophages. Histological examination showed increased collagen levels with fibers deposited neatly on the skin and a lower rate of necrotic tissue [35].

## 4. Advantages of Nanocarriers for Topical Insulin Delivery

The topical administration of growth factors in chronic wounds, such as epidermal growth factor or platelet-derived growth factor, have shown favorable results in skin regeneration [67]. The degradation of growth factors in the wound bed is rapid and the length of time required for growth factors to react with the cells is not yet known [68].

Nanocarriers are small drug delivery systems in form of micelles, liposomes, and nanoparticles based on biological or synthetic materials that are vehicles for drugs offering protection against degradation. Growth factors are often unstable biomolecules, susceptible to temperature, light or ionic strength changes, leading to denaturation, and reduced biological activity [69]. Insulin is one of the cheapest growth factors available on the market, but its topical therapeutic use has been associated with a short half-life in vivo due to proteases in the wound bed. Hence, insulin encapsulation ensures proteolysis prevention, maintaining the amino acid chain and the three-dimensional protein arrangement [70]. The physicochemical properties of nanoparticles, such as size and association efficiency (AE), can be tailored, and nanoparticles features can be tuned to increase drug bioavailability and to modify the biological action [71]. In addition of presenting biocompatibility, bioadhesion, swelling, and antimicrobial activity characteristics, the nanoparticles are versatile to vast product applications, suitable for a material for wound healing [23]. Their small size and increased surface area offer occlusive properties and a moisturizing effect, as well as intracellular entry and a transfer facility throughout the skin layers [72]. The nanoparticles’ capacity to provide a controlled and sustained release, avoiding the need for a frequent administration, also allow the administration of lower doses, proving to be a valuable strategy for chronic wound treatment [70]. Overall, the encapsulation of insulin is an excellent approach to avoid rapid clearance and improve wound healing [73].

## 5. Nanocarrier Systems for Topical Insulin Delivery

**Figure 4:**
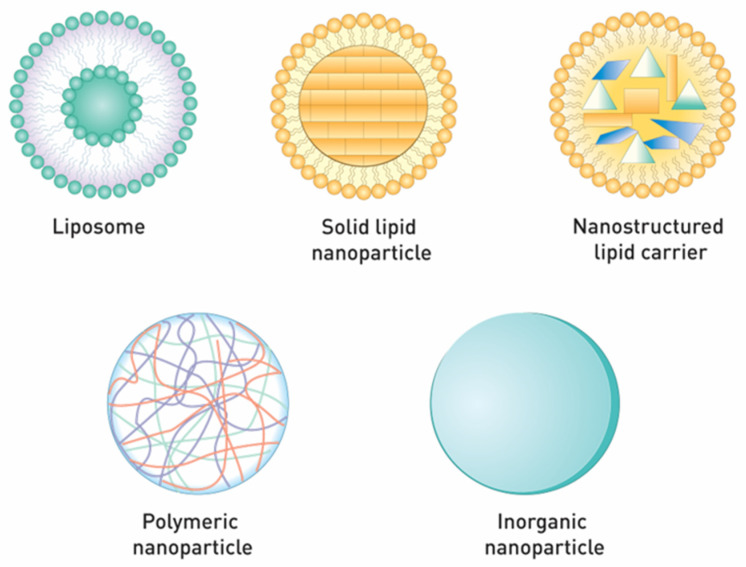
Types of nanoparticles for topical insulin delivery.

Insulin has shown promising results for cutaneous wound care, but its low stability in the wound bed is an important drawback. Thus, its encapsulation into nanocarriers has been suggested as a promising strategy [74]. A small amount of studies addressing the use of insulin-loaded nanocarriers for wound healing applications are reported. In this section, the different types of nanoparticles (Figure 4) under consideration for topical delivery of insulin are described. They are mostly from lipid, polymer, or inorganic origin, and are briefly summarized in Table 2.

### 5.1. Lipid-Based Nanoparticles

Lipid-based nanoparticles, including liposomes, solid lipid nanoparticles (SLN) and nanostructured lipid carriers (NLC) have been widely used for topical application due to their ability to release drugs in a sustained manner, displaying high tolerance, adequate skin absorption, and epidermal drug accumulation [75].

#### 5.1.1. Liposomes

Liposomes were the first lipid nanocolloids to be produced due to their easy production. They are formed by at least two lipophilic layers, which can load hydrophobic and hydrophilic drugs, with a dimension usually between 100 and 1000 nm [76]. Liposomes are typically composed of phospholipids consisting of a polar head and two nonpolar chains. Because of their amphipathic features, when in contact with water membranes, hydrophilic heads interact with the aqueous solution and hydrophobic tails interact with each other [77]. Hydrophilic drugs may be incorporated into the interior domain of the liposome, including genetic material for delivery of non-viral gene vectors, avoiding viral particle toxicity [78]. Hydrophobic active ingredients may be integrated into the coupled lipid layer, while ligands may be attached to the outer layer. The incorporation of polyethylene glycol (PEG) into the outer layer of the liposome provides protection from rapid degradation and allows sustained and controlled release [79]. Due to the presence of lipid bilayers that mimic the cell membrane of the human body, liposomes have been extensively used for topical drug administration [80]. Wound dressings made of non-woven gauzes impregnated with liposomes have been used showing drug release and more accurate management of chronic topical inflammation [81]. Conversely, liposomes have revealed poor long-term stability leading to reduced encapsulation efficiency, low reproducibility, heterogeneous size distribution, and sudden and uncontrolled drug release during storage [82]. Liposomes can easily sediment, aggregate, lose shape when intensely shaken or pressed, and accumulate under the continuous skin administration as the epidermal clearance of the drug is slower [83].

Dawoud and co-workers (2018) prepared insulin-loaded liposomes by employing various lipids and incorporating them into a chitosan-based spray. The particle size of the different liposomal insulin formulations ranged from 0.7 up to 2.9 μm, depending on the use of cholesterol, as this lipid increases the vesicle diameter. Insulin encapsulation efficiency was between 37% and 84% due to the preparation technique and sonication approach that decreased the amount of loaded drug. Through Franz diffusion cells, the insulin dispersion and the optimized formulation expressed a prolonged release of 6 h and up to 24 h, respectively [84]. The healing potential of insulin-containing liposomes was evaluated by the same group, which impregnated a chitosan gel with insulin-loaded liposomes and evaluated the clinical outcomes in non-diabetic patients [85]. Liposomal insulin in aqueous dispersion was stable for 6 months at 4 °C, and its release was sustained for 24 h. The clinical trial was coordinated in 15 patients over 8 months, in which liposomal insulin showed wound closure 16 times faster than the control group, with less erythema and no lowering blood sugar.

#### 5.1.2. Solid Lipid Nanoparticles and Nanostructured Lipid Carriers

Solid lipid nanoparticles (SLN) were developed to overcome liposome limitations. SLN consist of small carriers formed by solid lipids at body and room temperature [84]. They have been used as drug delivery platforms for topical administration due to their biocompatibility, biodegradability, increased drug permeation through the skin, easy preparation, and viability of large scale production [85]. Nanostructured lipid carriers (NLC) are composed of a mixture of solid and liquid lipids. They are second generation lipid nanoparticles generated to improve drug loading, provide prolonged drug release, and reduce drug expulsion during storage related to solid lipid nanoparticles [86]. SLN and NLC have been used on injured skin, as they can be formulated with non-irritant lipids [87] and have shown sustained release of occlusive formulations, promoting drug penetration into the skin [88]. There are no relevant studies addressing the delivery of insulin using SLN and NLC, but both lipid nanocarriers have shown their ability to topically deliver growth factors in a sustained and controlled manner, so they may be considered as potential carriers for topical delivery of insulin.

Previously, Gainza et al. delivered the recombinant human epidermal growth factor (rhEGF) in SLN and NLC to wounds, obtaining an encapsulation efficiency (EE) of SLN-rhEGF and NLC-rhEGF of 74% and 96%, respectively. The in vitro studies were conducted in HFF fibroblasts, HaCaT keratinocytes, and Balb/c 3T3 fibroblasts, showing the cell proliferation effect of rhEGF compared to empty nanoparticles as well as the loading of rhEGF into lipid nanoparticles enhanced the cell division activity compared with that derived with cleared rhEGF (*p* < 0.05). The efficacy of the SLN-rhEGF and NLC-rhEGF was probed in a full-thickness excisional wound in diabetic mice, showing improved skin reparation. The topical administration of 10 and 20 μg of SLN-rhEGF and 10 and 20 μg of NLC-rhEGF exhibited decreased wound area in comparison to empty lipid nanoparticles and untreated control (*p* < 0.05). Furthermore, results were not significantly different between the 10 μg and 20 μg doses [89].

In another study, the same authors evaluated the efficacy of rhEGF-loaded NLC in a full-thickness excision wound model in pigs. The group treated with 20 μg of topically applied rhEGF-NLC twice weekly showed a faster rate of healing, in contrast to the group treated with 75 μg of free rhEGF and unloaded NLC. In addition, rhEGF-loaded NLC stimulated angiogenesis, cell migration and proliferation, and collagen deposition. No significant levels of plasma rhEGF were found, showing evidence of no systemic absorption having occurred [90].

### 5.2. Polymer Nanoparticles

Polymers are macromolecules composed of repeated monomer units, in which their chain length depends on the number of the monomer units as well the molecular weight of these individual monomers. The polymeric materials may come from natural and synthetic origin being used separately or blended together for many applications, including wound healing [91].

#### 5.2.1. Natural Polymers

Chitosan, hyaluronic acid, cellulose, silk fibroin, gelatin, and collagen are polymers of natural origin widely used to obtain nanocarriers. Chitosan is a chitin-derived polymer found in the insect and crustacean exoskeleton, composed of D-glucosamine and N-acetyl-D-glucosamine. This polysaccharide is widely used in topically applied vehicles owing to its biocompatibility, biodegradability, microbicidal activity, low toxicity, and angiogenesis promoter [92]. Moreover, chitosan is able to grant cell adhesion and proliferation [93] and cell activation such as fibroblasts, leukocytes, macrophages, transforming growth factor β1 or platelet-derived growth factor display [94]. Furthermore, chitosan-based materials mainly show positive charge, offering mucoadhesiveness, hemorrhage control, and wound healing stimulation [95].

Ishihara et al. studied the effect of a chitosan hydrogel on full-thickness incision wounds on the back of mice [96]. The hydrogel was physically cross-linked using ultraviolet light irradiation. Round-shaped wounds, with approximately 100 mm2, were performed on the back of C57BL/6 mice with the help of a scalpel and scissors. Results showed rapid re-epithelialization, granulation tissue development, and hemostatic effect, reaching 90% of skin incision closure in one week, in contrast to the control group (*p* < 0.05). Ehterami et al. formulated chitosan nanoparticles loaded with insulin using ionotropic gelation to be incorporated into an electrospun poly(ε-caprolactone)/collagen. The wound dressing was applied onto 1.5 × 1.5 cm^2^ full-thickness wounds on the back of Wistar rats. Their skin backs were excised using a surgical knife, and after 2 weeks of treatment, wounds showed both epidermal and granulation tissue formation with a scab, while control rats exhibited approximately 60% of wound length. Furthermore, Masson’s trichrome staining results showed foremost collagen synthesis and maturation, in contrast to the control group [97]. Although chitosan has been considered safe for wound dressing applications [98], chitosan-based formulations are not yet acceptable for scale-up production due to limited production reproducibility [99].

Hyaluronic acid is an important component of all connective tissues and due to its biodegradability, moisturizing ability, cellular motility, proliferation promotion, and ability to induce inflammatory signals [100], it is regularly used in wound healing to promote tissue repair. Hyaluronic acid is involved in all healing phases of wounds and contributes to the reduction of reactive oxygen species (ROS) at the wound site [101]. Hirakura et al. developed a hyaluronic acid-based hydrogel involving nanogels formed from cholesteryl group with pullulan content and insulin for in vitro and in vivo release studies. The authors observed sustained and controlled release of insulin due to the crosslinking degree without bioactivity loss [102]. Recently, Nyman and co-workers conducted a clinical study in 10 patients to explore the outcome of intradermal hyaluronic acid administration concerning keratinocyte stimulation and dermal re-epithelization during incisional healing. Two volunteer groups were formed; one group was treated with hyaluronic acid injections and the other treated with saline injections. The hyaluronic acid injections-treated group showed 10-fold increased protein expression involved in wound healing and an increased wound healing rate compared to saline-treated wounds. After 24 h, 90% of the hyaluronic acid injections-treated group exhibited restoration of the epithelium, in contrast to saline-treated wounds that remained unchanged [103].

Alginate is a polysaccharide obtained from brown algae, consisting of different amounts of (1–4)-linked β-D-mannuronic acid (M) and α-L-guluronic acid (G). These G residues favor gelation in the presence of divalent ions, enabling the formation of nanoparticles. Alginate and its salts present hemostatic and regenerative characteristics that have been used for wound dressings, stimulating cell migration, enhancing angiogenesis, and decreasing proinflammatory cytokine levels in chronic wounds. Due to their hydrophilic nature, alginate dressings maintain a moist environment, absorbing wound exudate and restricting bacterial infections at the wound site [104]. In 2010, Borselli et al. formulated an injectable alginate gel containing both insulin-like growth factor-1 (IGF-1) and VEGF and delivered it in ischemic mice. Only IGF-1 treatment reduced the apoptosis process and increased cell activation and proliferation, leading to the considerable reconstruction of muscle fibers at the wound site. Nevertheless, IGF-1 showed an in vitro release of approximately 80% in the first 24 h, in contrast to VEGF, which presented a sustained release profile showing an in vitro release of approximately 40% in the first 24 h [105]. Although natural polymers offer biocompatibility and biodegradability, only with synthetic polymers is there structure control, providing tailorable properties.

#### 5.2.2. Synthetic Polymers

Polycaprolactone (PCL), polyethylene glycol (PEG), poly(lactic acid) (PLA), poly(glycolic acid) (PGA), and poly(lactic-co-glycolic acid) (PLGA) are synthetic polymers that have been used in regenerative medicine [106]. PLGA, a copolymer formed by glycolic and lactic acid has been used to produce nanoparticles due to its biodegradability and sustained release properties [107,108,109]. During its degradation, lactate is released, promoting the formation of new blood vessels [108].

Hrynyk et al. encapsulated crystallized insulin in PLGA carriers for topical administration to improve wound healing. Insulin biological activity showed activation of insulin receptors in cell culture-based phosphorylated AKT and triggered HaCaT cell migration using a cell scratch assay over a period of 3 weeks, versus the insulin-free control [109]. Later, in 2012, Hrynyk et al. encapsulated insulin into PLGA carriers to be incorporated into an alginate-containing PEG sponge. This sponge dressing formed a hydrogel, providing protection and a moist environment needed for the wound healing process. Sustained release profile was maintained up to 21 days, demonstrating migration rates of human epidermal keratinocytes for 10 days, compared to 10^−7^ M insulin solution [110]. In 2018, Abdelkader et al. considered the delivery of insulin-loaded PLGA nanoparticles with varying amounts of PEG embedded in a PVA-borate hydrogel. Although the authors found that variation of PEG concentration had low impact on wound closure rate, they observed faster cell proliferation on human fibroblasts (Hs27) and keratinocytes (HaCaT) in cell scratch assay following the addition of nanoencapsulated insulin, compared to free insulin [111]. In another approach, insulin encapsulation in PLGA nanoparticles was developed by a modified double emulsion solvent evaporation technique and incorporated in a poly(vinyl alcohol)-borate hydrogel to evaluate wound healing in healthy and diabetic rats. The insulin-loaded PLGA nanoparticles had 34 μg/mg of insulin and a diameter of 203 nm [74]. Both healthy and diabetic mice treated with insulin-loaded PLGA nanoparticles incorporated into the PVA-borate hydrogel exhibited faster wound recovery than healthy and diabetic mice receiving free insulin incorporated into the hydrogel (*p* < 0.001). After 10 days, the deviation between the percentage wound injury index of unloaded insulin was 12% in comparison to the control, whereas the deviation between the percentage wound injury index of insulin-PLGA nanoparticles was 30% in contrast to control.

### 5.3. Inorganic Nanoparticles

The application of inorganic nanoparticles for topical insulin delivery is neglectable. However, in this section, different inorganic nanoparticles are addressed with a foreseen potential for that purpose. Metal nanoparticles are submicron particles of metallic (silver, gold, cerium, or titanium) and magnetic (iron oxide) origin [112]. Silver nanoparticles are easily obtained by safe and inexpensive methods, and can be used in dressings, exhibiting antibacterial activity against a wide range of bacteria without developing microbial resistance [113]. Both sulfadiazine and silver nitrate have been shown to attenuate mast cell infiltration as well as cytokine and lymphocyte levels [114]. In 2007, Tian and co-workers subjected BALB/C mice to deep partial thickness thermal burns to evaluate healing time using silver dressings. BALB/C mice burns treated with silver nanoparticles healed in 27 days compared to mice treated with silver sulfadiazine and saline taking an average of 37 and 35 days to heal the wounds, respectively [115]. Liu et al. (2010), conducted a study to evaluate the effect of silver nanoparticles and silver sulfadiazine on dermal contraction, and epidermal re-epithelialization in a full-thickness excisional wound model on mice. AgNPs-treated wounds revealed faster closure, a higher rate of fibroblast differentiation into myofibroblasts, and increased proliferation and deposition of keratinocytes compared to silver sulfadiazine-treated wounds [116]. Moreover, silver nanoparticles are highly reactive due to negative surface charge, providing a strong interaction with amine or thiol groups [117]. Kaur et al. coated silver nanoparticles with insulin for the treatment of diabetic wounds. The study was performed on human epidermal keratinocyte cells and male Wistar rats in which the authors found that pro-inflammatory (TNFα, IL-6) and anti-inflammatory (IL-10) cytokines levels were regulated in the wound, contributing to rapid healing [118].

In 2010, Rakhmetova and co-workers described a study of an ointment containing copper nanoparticles for wound healing in mice. Copper nanoparticles were modified using oxygen vapors, air, and water and developed by condensation at high temperatures. The authors observed that the ointment containing 100 nm diameter copper nanoparticles, modified with O_2_ and having 95% of crystalline copper content, increased wound remodeling, compared to the ointment without copper nanoparticles [119].

Chigurupati et al. observed that the topical application of cerium oxide nanoparticles (nanoceria) enhanced the healing of full-thickness dermal wounds. Cell proliferation was determined in human keratinocyte cells and mouse fibroblasts, in which an increased cell proliferation rate was found in the presence of 1 and 10 µM nanoceria. The authors also found that the nanoceria decreased oxidative stress, allowing regenerative tissue preservation, thus increasing the proliferation of fibroblasts, keratinocytes, and endothelial cells [120].

In 2014, Sankar and collaborators prepared, by green synthesis, titanium dioxide nanoparticles (TiO2.NPs) from the leaf extract of Origanum vulgare. Healing activity was examined using a full-thickness excision wound model in Albino rats. Wound contraction was measured by microscopic examination of the granulation tissue and by evaluating the protein expression. Animals treated with topical TiO2.NPs showed no signs of hemorrhage or infection, and wound retraction was observed by day 4. On the 12th day, SDS-PAGE analysis revealed differentially expressed proteins on TiO2.NPs treated animals, showing increased density on the 25, 55, 75, and 150 kD bands, in contrast with the control group. The rats treated with topical saline solution following histopathological analysis showed low fibroblast levels and collagen deposition when compared to TiO2.NPs-treated animals, demonstrating the therapeutic efficacy of the nanoparticles [121].

Gold nanoparticles are also effective for wound care, as gold is a reliable drug carrier. Taking advantage of the specific gene expression for skin repair, Randeria et al. associated gold nanoparticles with siRNA-based spherical nucleic acids (SNAs) to inhibit ganglioside-monosialic acid 3 synthetase (GM3S) overexpression, restricting insulin resistance and hindering wound healing in diabetic patients. The study conducted in cultured mouse keratinocytes showed stimulation of both insulin and insulin-like growth factor-1 (IGF1) receptors under normal and hyperglycemic conditions, as well as an increase of keratinocyte migration and proliferation, in contrast to control. Topical administration of spherical nucleic acids conjugated to AuNPs in full-thickness wounds in diabetic mice caused more than 80% reduction in GM3S synthesis at the wound site, and within 12 days diabetic wounds were completely closed, whereas only half of the control group wounds exhibited recovery [122]. Currently, gold nanoparticles have been produced using plants with antidiabetic properties. Ponnanikajamideen et al. developed, through green synthesis, gold nanoparticles from the *Chamaecostus cuspidatus* insulin plant to analyze the wound healing and hypoglycemic effect. Male Wistar rats were divided into four different groups for the wound-healing test: control double distilled water, vehicle control H_2_O, green synthesized Gold NPs, and plant extract. After the 4th week of post wounding, the authors observed that gold NPs and plant extract groups exhibited a higher healing rate and a prolonged anti-inflammatory and antioxidant effect over time, when compared to both control groups treated with double distilled water and vehicle control H_2_O [123].

Silica or silicon dioxide nanoparticles have been used for theragnostic applications due to their versatile properties such as easy production, adjustment, low toxicity, molecular stability, and targeted delivery [124]. Quignard and co-workers analyzed the wound healing process, by applying 50 and 100 μM of silica nanoparticles in an in vitro wound healing assay using human dermal fibroblasts (CCD-25SK). After 3 days of treatment, the authors observed that both concentrations stimulated cell viability and proliferation versus the control group (*p* < 0.05) [125].

## 6. Pipeline Products

Despite the many studies over the years suggesting the potential benefits of insulin-loaded nanocarrier systems for wound healing, no products are commercially available in the market. This is due to the need for further studies to gain more knowledge about safety and effectiveness of these topical delivery systems [127]. Notwithstanding, several commercially available products have emerged with the use of growth factors for wound healing, thus insulin may also follow this path.

Regranex gel (Healthpoint Biotherapeutics, Fort Worth, TX, USA) contains recombinant human platelet-derived growth factor. Regranex has been approved by the FDA for the prevention of lower-limb amputations in the cases of diabetic foot ulcers (DFUs) [128]. Patients with diabetic wounds treated with topical platelet-derived growth factors experienced around a 40% wound closure increase, compared to control group [129]. Fiblast^®^ Spray commercialized in Japan consists of recombinant fibroblast growth factor (rhbFGF) for the treatment of leg ulcers and burns [130].

Hayashida et al. showed that pediatric patients suffering from second-degree burns and treated with bFGF had decreased scarring and rapid healing, compared with the controls [131]. Other commercially available approaches to tissue regeneration are Easyef^®^, Regen-D™ 150, and Heberprot-P^®^. All of these approaches are based on recombinant human epidermal growth factor (rhEGF), effective in treating DFUs, pressure ulcers, vascular ulcers, and chronic leg ulcers. In particular, Regen-D™ 150 is a gel composed of 150 μg/g rhEGF, being topically applied to DFUs twice a day [70]. Heberprot-P^®^ includes 75 μg rhEGF, being injected to the wound bed three times per week. However, additional studies are needed to compare the safety and efficacy of rhEGF with other growth factors [132].

Johnson & Johnson has developed Promogran for topical wound therapy in the form of sterile, lyophilized, oxidized regenerated cellulose and collagen, deactivating the wound bed proteases and protecting the growth factors that are present in the wounds. Veves et al. conducted a randomized, prospective, controlled clinical trial, determining that Promogran was more effective in treating DFUs of less than 6 months compared with the wet gauze dressing used [133]. Recently, homologous platelet-rich plasma (PRP) was used for the treatment of chronic wounds, as thrombin activates platelets releasing mitogenic and chemotactic factors throughout the wound healing process. For this reason, Nuo Therapeutics, Inc. (Gaithersburg, MD, USA) developed the AutoloGel™ indicated for the treatment of chronic wounds being topically applied up to twice a week [134]. Shortly after, the same company launched Aurix™, a hematogel obtained from the patient’s own plasma and platelets to stimulate skin recovery [135].

In 2016, the Praxis Biopharma Research Institute (Álava, Spain) patented a nanoparticle delivery system for wound healing with SLN and NLC loading epidermal growth factor (rhEGF) and cathelicidin antimicrobial LL37 peptide, present at deficient levels in chronic ulcers. The researchers found a sustained release of the loaded rhEGF and a higher in vitro proliferation rate of fibroblasts than unloaded rhEGF [136]. Among the several products considered for topical administration, silver nanoparticles are often used in creams, gels, or dressings due to their effectiveness in hospital-acquired infections caused by antibiotic resistant bacteria, and local therapy of infected wounds [137]. Moreover, AgNPs have anti-inflammatory properties and collagen control regulation, inducing its proper arrangement during wound closure. Although the mechanism is still unknown by which silver nanoparticles act on collagen, in several clinical cases where AgNPs-containing dressings were studied, results suggest that AgNPs improved epithelial regeneration, compared with current commercially available wound therapy dressings [138].

## 7. Toxicity Concerns

Nanoparticles for drug delivery offer protection of the active compounds, biodegradability, and sustained release, providing treatments that are safer and more efficient [139]. However, their size, high surface area, surface reactivity, form, chemical composition, and time of residence are the physicochemical features that can drive nanoparticles to present toxicity. Nanometer-sized particles have strong exposure to fluids and tissues due to their larger contact surface and greater biological reactivity, possibly affecting the regulation cell mechanisms [140]. Schneider et al. reported that it is necessary to address the nanoparticles size and their mechanism of action on the skin for wound healing [141]. The resulting by-products may accumulate in cells inducing transmutations, triggering cell hemostasis disturbance and oxidative stress formation [142].

Upton et al. conducted a clinical trial composed of 30 patients suffering from pressure ulcers, diabetic foot ulcers, and venous leg ulcers on which they only observed two cases of adverse events associated with insulin-like growth factor (IGF) and epidermal growth factor (EGF) when 132.5 µg of vitronectin was topically applied. In one case, burning after dressing application was reported, and in the other, an itching sensation at the ulcer site was reported [143]. Dhall et al. also observed in vivo that topical insulin-loaded PLGA microparticles delivery decreases levels of reactive oxygen species. The activity of superoxide dismutase (SOD) was measured at 450 nm by the reduction of tetrazolium salts. The authors found that insulin therapy decreased SOD and H_2_O_2_ levels during the first 3 days of healing [35].

The biodegradable polymeric nanoparticles, such as poly-ε-caprolacton (PCL), PLA, and PLGA, have been preferred as drug carriers due to their biocompatibility, biodegradation, non-immunogenicity, and non-toxicity [35]. Several authors have expressed the safety of gold nanoparticles at the cellular level, while others have indicated DNA damage during cell division and immune system activity, demonstrated by both in vitro and in vivo analysis [144]. Agryria, a bluish-gray tinge in the skin, as well as cellular toxicity and impaired fibroblast repairment processes have been correlated with the continued use of silver nanoparticles [145]. Nevertheless, the toxicity of AgNPs can be decreased by using green synthesis with natural capping agents to remove hazardous chemicals [146]. In the case of silica nanoparticles, Ryu et al. showed that percutaneous application of 20 nm silica particles for 3 months in Sprague Dawley rats did not cause skin or internal organ toxicity [147]. Another example is impaired cell function on human dermal fibroblasts caused by titanium dioxide nanoparticles. Pan et al. formulated TiO2.NPs coated with polymer molecules, namely proanthocyanidins/poly(methyl-vinylether/maleic acid) (30% *w/w*) and triethoxysilylethyl poly(dimethylsiloxyethyldimethicone) (5% *w/w*). Due to this process of coating, enhanced human dermal fibroblasts protection and reduced reactive oxygen species was observed through flow cytometry [148].

## 8. Conclusions

Wound healing is a biological process resulting from the restoration of the integrity of the skin barrier. The use of nanotechnology to accomplish topical delivery of insulin has demonstrated to be effective for wound healing. The use of nanoparticles to deliver insulin to wounds increases their stability, while providing adhesion to the mucosal surfaces and preserving the sustained release enabling safer treatments. Insulin has been shown to have the ability to reduce inflammation, decrease oxidative damage, promote tissue neogenesis, as well as promote collagen deposition and maturation, at a lower cost than other repairing growth factors. Despite promising studies developed so far, no formulation has yet been launched on the market. Additional studies are needed for a better understanding of the bioactivity and toxicity of nanoparticles applied to wounds and to ensure insulin bioavailability in the skin, in order to achieve a safe and effective insulin nanotechnology treatment for wound care. It is expected that this review may be a contribution to leverage the development of therapeutically effective and safe nanocarrier systems for topical insulin delivery.

## Figures and Tables

**Table 1:** Types of chronic wounds and its main features.

Wound Type	Etiology	Microenvironment	Bacterial Environment	Average Healing Time	Ref.
Pressure ulcers	spinal cord injury paralysis senility substance abuse malnutrition stroke multiple sclerosis	shear stress friction high humidity temperature tissue atrophy	*Enterococcus Staphylococcus Pseudomonas* *Serratia* *Proteus* *Corynebacterium Staphylococcus Anaerococcus* *Bacteroides*	2 weeks	[24,25,26]
Diabetic foot ulcers	hyperglycemia poor circulation neuropathy wounded feet	ischemia infection foot deformity callus trauma pressure	*Enterococcus* *Pseudomonas Streptococcus* *Serratia* *Staphylococcus Anaerococcus Corynebacterium Pseudomonas*	6 months	[27,28,29]
Venous leg ulcers	age obesity physical inactivity trauma deep vein thrombosis phlebitis	chronic venous hypertension vein walls structural failure valve system failure	*Staphylococcus Serratia* *Streptococcus Pseudomonas Corynebacterium Staphylococcus Bacteroides*	4 months	[30,31]

**Table 2:** Brief summary of nanoparticles encapsulating insulin used for wound healing applications.

Type of Nanoparticles	Material	Size (nm)	PdI	Zeta Potential (mV)	In Vitro Model	In Vivo Model	Wound Decrease (%)	Ref.
Liposomes	Phosphatyl choline, cholesterol, chitosan	184–701	-	−22.9 to −18	-	Human	40	[85]
Polymeric nanoparticles	Chitosan, poly (ε-caprolactone)/collagen	256	0.23	17.89	L929 cells	C57BL/6 mice	~63	[97]
	PLGA, PEG	297	0.15	−3.94	HaCat, human fibroblasts	-	-	[110]
Inorganic nanoparticles	Silver	10	-	-	Mouse embryo fibroblast cell line (BALB/3T3; clone A31)	C57BL/6N mice	~31	[116]
	Silver	42	-	−15.1	Human epidermal keratinocyte cells (HEKa)	Wistar rats	~73	[118]
	Copper	119	-	-	-	Mice of the SHK line	~52	[119]
	Nanoceria	5	-	-	HMEC-1 cells	C57BL/6 mice	-	[120]
	Titanium dioxide	341	-	−27.3	-	Wistar Albino rats	94	[121]
	Gold	50	-	-	-	Wistar rats	97	[126]
	Silica	10	-	−30 to +23	CCD-25SK cells	-	-	[125]

## Data Availability

Not applicable.
